# Identifying premenopausal patients with early-stage hormone receptor–positive breast cancer at minimal risk of distant recurrence by breast cancer index

**DOI:** 10.1016/j.breast.2026.104714

**Published:** 2026-01-29

**Authors:** Ruth M. O'Regan, Yue Ren, Yi Zhang, Gini F. Fleming, Prudence A. Francis, Olivia Pagani, Barbara A. Walley, Roswitha Kammler, Patrizia Dell’Orto, Giuseppe Viale, Sherene Loi, Marco Colleoni, Kai Treuner, Meredith M. Regan

**Affiliations:** a Department of Medicine, University of Rochester, Rochester, NY, USA; b IBCSG Statistical Center, Division of Biostatistics, Dana-Farber Cancer Institute, Boston, MA, USA; c Biotheranostics, A Hologic Company, San Diego, CA, USA; d The University of Chicago Medical Center, Chicago, IL, USA; e The Sir Peter MacCallum Department of Medical Oncology, The University of Melbourne, Parkville, VIC, Australia; f Department of Medical Oncology, Peter MacCallum Cancer Centre Melbourne, VIC, Australia; g St Vincent's Hospital, Melbourne, Australia; h Breast Cancer Trials Australia & New Zealand, Newcastle, Australia; i Geneva University Hospitals, Geneva, Switzerland; j Lugano University, Lugano, Switzerland; k Swiss Group for Clinical Cancer Research (SAKK), Bern, Switzerland; l University of Calgary, Calgary, AB, Canada; m Canadian Cancer Trials Group (CCTG), Queen's University, Kingston, ON, Canada; n Translational Research Coordination, International Breast Cancer Study Group, ETOP IBCSG Partners Foundation, Bern, Switzerland; o IBCSG Central Pathology Office, Milan, Italy; p Department of Pathology and Laboratory Medicine, IEO European Institute of Oncology IRCCS, Milan, Italy; q Division of Cancer Research, Peter MacCallum Cancer Centre, Melbourne, Australia; r Division of Medical Senology, IEO European Institute of Oncology, IRCCS, Milan, Italy; s Harvard Medical School, Boston, MA, USA

**Keywords:** Breast cancer, Breast cancer index, Endocrine therapy, Premenopausal

## Abstract

**Background:**

An adjusted Breast Cancer Index (BCI) model with an additional cutpoint identified postmenopausal women with hormone-receptor-positive node-negative disease at minimal (<5%) risk of distant recurrence (DR) within 10 years.

**Methods:**

2025 premenopausal patients with hormone-receptor-positive node-negative breast cancer, randomized to adjuvant endocrine therapy in SOFT and TEXT (35.6% and 40.4% received adjuvant chemotherapy, respectively), previously had BCI assessed. The additional BCI cutpoint re-classified a subset of the low-risk group into minimal-risk; those in intermediate- or high-risk groups were unchanged. The 10-year DR was estimated by Kaplan-Meier method.

**Results:**

The adjusted BCI model re-classified 17.8 % and 19.6 % of node-negative disease in SOFT and TEXT into BCI minimal-risk groups; 43.2 % and 38.3 % remained classified in low-risk groups, respectively. In SOFT, the estimated 10-year DR was 2.3 % (95 %CI 0.9–6.0 %) and 4.1 % (95 %CI 2.6–6.5 %) in the minimal-risk and revised low-risk groups, respectively. In TEXT, the estimated 10-year DR was 2.0 % (95 %CI 0.7–6.2 %) and 4.6 % (95 %CI 2.8–7.7 %) in the minimal- and low-risk groups, respectively.

**Conclusions:**

This study confirmed prognostic ability of the minimal-risk BCI cutpoint to classify patients estimated to have minimal-risk of distant recurrence within 10 years among premenopausal patients treated for hormone-receptor-positive node-negative breast cancer, providing relevant information for personalizing adjuvant endocrine therapy.

**Soft:**

(clinicaltrials.gov NCT00066690)

**Text:**

(clinicaltrials.gov NCT00066703)

## Highlights

• Additional BCI cutpoint identified postmenopausal women with HR + N0 disease at minimal (<5 %) 10-yr distant recurrence risk
• Approx. 60 % of premenopausal patients with HR+ N0 breast cancers enrolled in SOFT and TEXT were classified as BCI low risk.
• Approx. 20% of these premenopausal patients were re-classified as minimal risk using the additional cutpoint.
• The BCI minimal-risk subset had estimated 10-year distant recurrence risk of approx. 2% (upper 95%CI of 6%).

## 1 Introduction

The randomized Suppression of Ovarian Function Trial (SOFT) and Tamoxifen and Exemestane Trial (TEXT) provided evidence for the use of ovarian function suppression (OFS) with either tamoxifen or with the aromatase inhibitor exemestane as part of standard of care adjuvant endocrine therapy for premenopausal women with hormone-receptor-positive (HR+) early breast cancer [1,2]. Although treatment with an OFS-containing regimen improves outcomes [[3], [4], [5]], OFS-containing regimens are associated with greater adverse effects than tamoxifen alone that must also be considered when making treatment decisions [3,6].

Gene expression tests are increasingly used in clinical settings to support adjuvant therapy decision-making. The Breast Cancer Index (BCI) is validated to provide individualized risk of distant recurrence for patients with early-stage HR+ breast cancer [[7], [8], [9], [10], [11], [12]]. In premenopausal patients with node-negative breast cancer enrolled in SOFT and TEXT, the BCI continuous score and its classification into 3 risk-groups were shown to be prognostic for distant recurrence over a median follow-up of 12 and 13 years [13,14]. Approximately 60 % of patients with node-negative cancers were classified as BCI low-risk, for whom the estimated risk of distant recurrence within 12 years was approximately 4 %, compared to 10 % and 15 % among those classified as intermediate- or high-risk, across all treatment groups combined.

An additional BCI “minimal-risk” cutpoint was selected and validated in cohorts of postmenopausal patients who had no or endocrine-only adjuvant treatment enrolled in the Stockholm STO-3 and Tamoxifen and Exemestane Adjuvant Multinational (TEAM) trials and in a cohort from the Netherlands Cancer Registry [15,16]. In those studies, 14 %–22 % of postmenopausal patients were re-categorized as BCI minimal risk, with 10-year risk of distant recurrence less than 5 % irrespective of treatment with endocrine therapy, and in the absence of chemotherapy, supporting the use of BCI minimal risk for de-escalation of adjuvant endocrine therapy in postmenopausal women. The current study evaluated the adjusted BCI model re-classification as minimal-risk rather than low-risk, among premenopausal patients with HR+ node-negative breast cancer in SOFT and TEXT, to assess its prognostic value for supporting decision-making about OFS-containing adjuvant endocrine therapy.

## 2 Methods

### 2.1 Patients and study design

The study designs and methods for the phase 3 SOFT and TEXT randomized clinical trials were previously published [17,18]. In SOFT, women with early-stage HR+ breast cancer were randomized 1:1:1 to receive 5 years of adjuvant tamoxifen alone, tamoxifen + OFS, or exemestane + OFS. Prior chemotherapy was optional. Patients were randomized within 12 weeks after definitive surgery (if no chemotherapy was planned), or within 8 months after finishing chemotherapy and confirming premenopausal status. Randomization was stratified by prior receipt of chemotherapy and lymph-node status.

In TEXT, premenopausal patients were enrolled within 12 weeks after definitive surgery and randomized 1:1 to receive 5 years of adjuvant tamoxifen + OFS or exemestane + OFS. Adjuvant chemotherapy was optional, initiated simultaneously with triptorelin. Randomization was stratified according to lymph-node status and planned use of adjuvant chemotherapy.

This analysis included patients with HR+ node-negative breast cancer from SOFT and TEXT for whom formalin-fixed paraffin-embedded (FFPE) tumor samples were available for RNA extraction and BCI was previously determined [13,14] (Supplemental Tables S1 and S2). The BCI translational studies were approved by the IBCSG Translational Working Group and reported following REMARK guideline.

### 2.2 BCI assay and cutpoints

FFPE primary tumor specimens were macrodissected by the IBCSG Central Pathology Office to enrich tumor content and RNA was extracted. As previously published [11,12], RT-PCR analyzed BCI gene expression in the resulting RNA. Total RNA was reverse transcribed, resulting cDNA was pre-amplified by PCR using the Thermo Fisher Scientific PreAmp Master Mix Kit, TaqMan PCR analysis was performed, and BCI calculated. The laboratory was blinded to clinical outcome.

The current BCI prognostic model categorized patients as low-, intermediate-, or high-risk of distant recurrence within 10 years, using previously-validated cutpoints of 5.1 and 6.5 [11,12]. The newly-developed cutpoint of 3.0 re-classified a subset of low-risk patients into a minimal-risk group, with others remaining in a revised low-risk group. The new BCI cutpoint of 3.0, together with the current cutpoints of 5.1 and 6.5, re-classified SOFT and TEXT patients as minimal- or low-risk, and those in intermediate and high-risk groups remained as previously classified [13,14].

### 2.3 Statistical considerations

The primary objective assessed the prognostic performance of the minimal-risk group in premenopausal patient cohorts of SOFT and TEXT with node-negative cancer. Analyses were separate by trial to provide replication of the assessment and because of differences in study designs including assigned and elected adjuvant treatments. The median follow-up was 12- and 13-years [1,2]. The primary endpoint was distant recurrence-free interval (DRFI), defined as the time interval from randomization until distant recurrence of breast cancer, or censored when last documented as free from distant recurrence. The Kaplan-Meier method estimated the 10-year DRFI for each cohort, plotted and reported as one minus Kaplan-Meier estimate with pointwise 95 % confidence interval (CI). The CIs were obtained using complementary log-log transformation of the survivorship function. Cox models, stratified by adjuvant chemotherapy and treatment assignment, assessed the prognostic performance specifically of the BCI new minimal-risk group vs revised low-risk group, in the context of the overall risk-group classification of the patient cohorts. Models adjusted for patient age, tumor grade and size, and HER2 status were also estimated to ensure consistency of results. In addition, Cox models stratified by treatment assignment estimated the linear relationship between continuous BCI and DRFI to report the probability of distant recurrence within 10 years (as one minus 10-year survivorship estimates) with pointwise 95 % CI. SAS statistical software, version 9.4 (SAS Institute Inc., Cary, NC), was used for the statistical analysis.

## 3 Results

Of the 1687 premenopausal patients in the SOFT BCI analysis cohort, 1110 (65.8 %) had node-negative (N0) breast cancers (Table 1). Among those with N0 cancers, 35.6 % had received chemotherapy prior to enrollment (14.7 % and 28.5 % of those classified as minimal- and low-risk, respectively; Supplementary Table S1). From the TEXT BCI analysis cohort, 915 (51.3 %) of 1782 premenopausal patients had N0 cancers; 40.4 % of patients initiated chemotherapy with OFS upon enrollment (27.4 % and 31.7 % of patients classified as minimal- and low-risk, respectively; Supplementary Table S2).

**Table 1:** Clinicopathologic characteristics of patients in the SOFT and TEXT BCI analysis cohorts with node-negative (N0) breast cancers^a^.

	SOFT N0						TEXT N0	
	All N0 in SOFT BCI Cohort		Tamoxifen		Exemestane + OFS or Tamoxifen + OFS		All N0 in TEXT BCI Cohort: Exemestane + OFS or Tamoxifen + OFS	
	n	%	n	%	n	%	n	%
*No. of patients*	*1110*	*100.0*	*383*	*100.0*	*727*	*100.0*	*915*	*100.0*
Chemotherapy^b^								
No	715	64.4	240	62.7	475	65.3	545	59.6
Yes	395	35.6	143	37.3	252	34.7	370	40.4
Age at randomization								
<35	92	8.3	37	9.7	55	7.6	65	7.1
35-39	173	15.6	71	18.5	102	14.0	133	14.5
40-44	315	28.4	104	27.2	211	29.0	327	35.7
45-49	382	34.4	126	32.9	256	35.2	313	34.2
50+	148	13.3	45	11.7	103	14.2	77	8.4
Tumor size								
≤2 cm	824	74.2	284	74.2	540	74.3	646	70.6
>2 cm	278	25.0	94	24.5	184	25.3	265	29.0
Unknown	8	0.7	5	1.3	3	0.4	4	0.4
Tumor grade^c^								
1	327	29.5	108	28.2	219	30.1	165	18.0
2	574	51.7	194	50.7	380	52.3	542	59.2
3	195	17.6	77	20.1	118	16.2	204	22.3
Unknown	14	1.3	4	1.0	10	1.4	4	0.4
HER2 status^c^								
HER2–	984	88.6	337	88.0	647	89.0	795	86.9
HER2+	100	9.0	35	9.1	65	8.9	115	12.6
Unknown	26	2.3	11	2.9	15	2.1	5	0.5

a In SOFT 1100 patients had N0 cancers of 1687 patients in the previously-analyzed BCI analysis cohort for whom FFPE tumor samples were available for RNA extraction and BCI determination [14]; in TEXT 915 patients had N0 cancers of 1782 patients in the TEXT BCI analysis cohort [15].

b Optional chemotherapy was prior to randomization in SOFT and after randomization in TEXT.

c Tumor grade and HER2 status were determined locally.

### 3.1 Prognosis of the adjusted BCI model in the SOFT N0 cohort

**Fig. 1:**
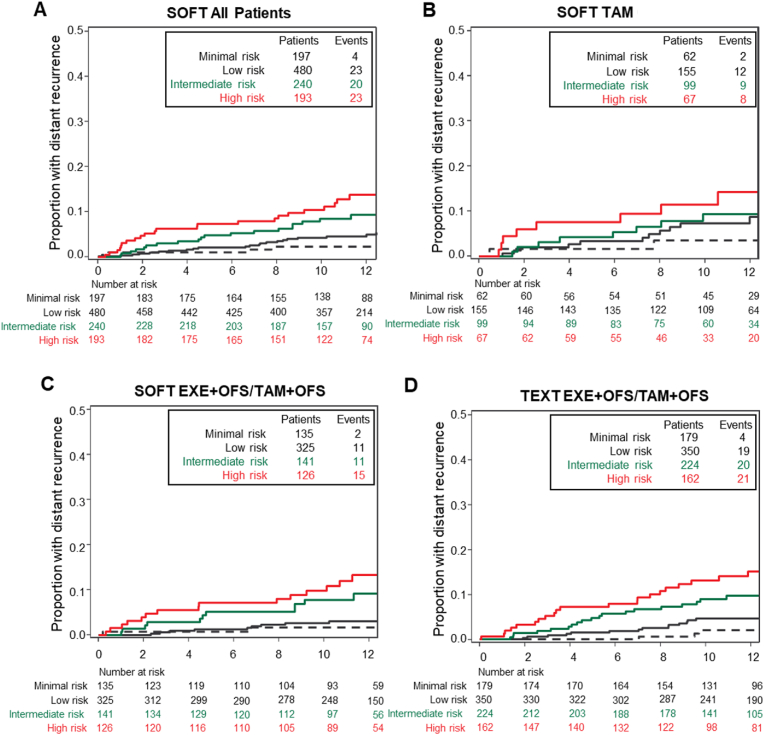
Kaplan-Meier estimates of distant recurrence-free interval in the SOFT and TEXT N0 cohorts according to the four BCI risk groups of the adjusted BCI model. The minimal-risk group is indicated by a dashed black line. (**A**) SOFT all patients; (**B**) SOFT assigned tamoxifen (TAM); (**C**) SOFT assigned exemestane (EXE) + OFS or tamoxifen + OFS; (**D**) TEXT all patients assigned exemestane + OFS or tamoxifen + OFS.

**Fig. 2:**
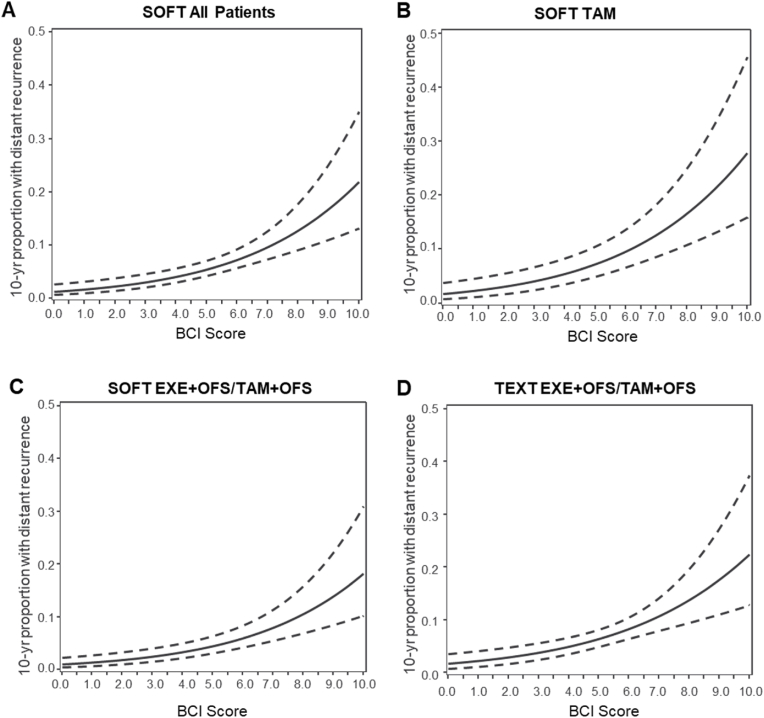
Relation of the continuous BCI score with estimated probability of distant recurrence within 10 years of randomization in the SOFT and TEXT N0 cohorts. (**A**) SOFT all patients; (**B**) SOFT assigned tamoxifen (TAM); (**C**) SOFT assigned exemestane (exemestane (EXE) + OFS or tamoxifen + OFS; (D) TEXT all patients. Values are one minus survivorship probabilities for distant recurrence-free interval (DRFI) at 10 years since randomization estimated from a Cox model, with pointwise 95 % CI. For the adjusted BCI model, the new cutpoint to differentiate minimal-from low-risk is 3.0; the current cutpoints for intermediate- and high-risk are 5.1 and 6.5.

The adjusted BCI model re-categorized 17.8 % of patients with N0 cancers as minimal-risk and 43.2 % remained as low-risk; there were 21.6 % and 17.4 % as intermediate and high risk based upon the current BCI model. The estimated 10-year risk of distant recurrence was 2.3 % (95 %CI, 0.9–6.0) among those patients re-classified as minimal risk, and for the revised low-risk group was 4.1 % (95 %CI, 2.6–6.5; HR vs minimal risk, 1.93 [95 %CI, 0.66–5.61]). For the intermediate- and high-risk groups the estimated 10-year distant recurrence was 8.4 % (95 %CI, 5.4–13.0) and 10.4 % (95 %CI, 6.8–15.9), respectively (Fig. 1A, Table 2). The estimated increasing function of 10-year distant recurrence risk in relation to the continuous BCI score is presented in Fig. 2A.

**Table 2:** Estimated distant recurrence probabilities at 10 years from randomization of the four risk groups of the adjusted BCI model in SOFT and TEXT N0 cohorts. Values are one minus Kaplan-Meier estimate of distant recurrence-free interval at 10 years since randomization (presented as percentages), with pointwise 95 % CI.

Cohort	Patients, no. ( %)	10-year Distant Recurrence, % (95 % CI)
*SOFT All*	*n = 1110*	
BCI minimal risk	197 (17.8)	2.3 (0.9–6.0)
BCI low risk	480 (43.2)	4.1 (2.6–6.5)
BCI intermediate risk	240 (21.6)	8.4 (5.4–13.0)
BCI high risk	193 (17.4)	10.4 (6.8–15.9)
*SOFT tamoxifen*	*n = 383*	
BCI minimal risk	62 (16.2)	3.5 (0.9–13.3)
BCI low risk	155 (40.5)	7.2 (4.0–13.1)
BCI intermediate risk	99 (25.8)	9.3 (4.7–17.8)
BCI high risk	67 (17.5)	11.4 (5.6–22.6)
*SOFT exemestane + OFS or tamoxifen + OFS*	*n = 727*	
BCI minimal risk	135 (18.6)	1.7 (0.4–6.6)
BCI low risk	325 (44.7)	2.7 (1.4–5.3)
BCI intermediate risk	141 (19.4)	7.9 (4.3–14.2)
BCI high risk	126 (17.3)	9.9 (5.7–16.8)
*TEXT All (exemestane + OFS or tamoxifen + OFS)*	*n = 915*	
BCI minimal risk	179 (19.6)	2.0 (0.7–6.2)
BCI low risk	350 (38.3)	4.6 (2.8–7.7)
BCI intermediate risk	224 (24.5)	9.0 (5.7–13.9)
BCI high risk	162 (17.7)	13.1 (8.5–19.7)

Abbreviations: BCI = breast cancer index; OFS = ovarian function suppression.

In the SOFT tamoxifen treatment group, BCI re-categorized 16.2 % and 40.5 % of patients as minimal- and low-risk, with estimated 10-year distant recurrence of 3.5 % (95 %CI, 0.9–13.3) and 7.2 % (95 %CI, 2.0–5.2), respectively (Fig. 1B–Table 2). In the SOFT exemestane or tamoxifen plus OFS treatment groups, 18.6 % and 44.7 % of patients were re-categorized by BCI as minimal- and low-risk with estimated 10-year of distant recurrence of 1.7 % (95 %CI, 0.4–6.6), and 2.7 % (95 %CI 4.0–13.1) respectively (Fig. 1C; Table 2). The 10-year estimates for the range of continuous BCI scores are shown in Fig. 2B and C according to assigned treatment regimen.

### 3.2 Prognosis of the adjusted BCI model in the TEXT N0 cohort

The adjusted BCI model re-categorized 19.6 % and 38.3 % of TEXT patients with N0 cancers as minimal- and low-risk; with 24.5 % as intermediate- and 17.7 % as high-risk. The estimated 10-year risk of distant recurrence was 2.0 % (95 %CI, 0.7–6.2) among the new minimal-risk group and for the revised low-risk group was 4.6 % (95 %CI, 2.8–7.7; HR versus minimal risk, 2.50 [95 %CI, 0.85–7.36]) (Fig. 1D–Table 2). The estimated 10-year risk of distant recurrence as a function of the full range of BCI scores is shown in Fig. 2D for the TEXT cohort.

## 4 Discussion

Studies have documented the efficacy of OFS for premenopausal patients with HR+ early breast cancer and additional benefits in terms of reducing risk of recurrence and death from breast cancer obtained with OFS relative to adjuvant endocrine therapy with tamoxifen alone [3,4]. Yet breast cancer guidelines do not recommend all patients to have OFS as part of adjuvant therapy [19]. Reliably identifying patients who are at very low risk of distant recurrence and may consider omitting OFS, especially for patients for whom the potential side effects of OFS treatment are of great concern, is an urgent clinical need.

The current analysis confirmed the clinical validity of an adjusted BCI model, intended to re-classify patients with currently considered BCI low-risk breast cancer into a subset at very low risk of distant recurrence, for premenopausal patients. The new BCI minimal-risk group was approximately 20 % of the premenopausal patients enrolled in SOFT and TEXT, with about 40 % remaining in the revised low-risk group. In the combined minimal risk groups of the SOFT and TEXT cohorts (n = 376), 8 distant recurrences were documented over median follow-up of 12–13 years, with estimated 10-year risks of distant recurrence of approximately 2 % and upper limits of the 95 % CIs of approximately 6 %. The estimated 10-year distant recurrence in the revised low-risk groups were about double those of the minimal-risk groups (i.e., hazard ratios around 2.0; 42 distant recurrences among 830 patients). Among patients assigned to tamoxifen alone in SOFT who remained in the revised BCI low-risk group, the estimated 10-year distant recurrence was 7.2 % (95 %CI 4.0–13.1 %), which may be considered as quite high for a young population with N0 cancers.

In SOFT a clinically-meaningful relative benefit (i.e., hazard ratio) of OFS combined with tamoxifen or exemestane compared to tamoxifen alone has consistently been observed across subgroups defined by individual clinical-pathologic features. For those subgroups associated with lowest risk of recurrence (e.g., N0, grade 1, tumor ≤2 cm), the relative benefit corresponds to small absolute benefits for distant recurrence of 1–2 % percentage points at 12 years [1]. The results of SOFT and TEXT have also been reported using a composite risk measure of multiple clinical-pathologic features, estimating the magnitude of absolute benefit of more intensive OFS-containing endocrine therapy regimens across a spectrum of underlying risk of distant recurrence, with minimal benefit among women at lowest risk [20]. The BCI minimal-risk group was defined based upon only gene expression analysis of tumor and developed independent of the SOFT and TEXT trials. The BCI minimal-risk group in the SOFT tamoxifen control group had 10-year distant recurrence estimate of 3.5 % (95 %CI 0.9–13.3; 2 distant recurrences among 62 patients over 12-year median follow-up) and 1.7 % (95 %CI 0.4–6.6) among patients assigned OFS + exemestane or OFS + tamoxifen. These estimates are consistent with prior SOFT and TEXT subgroup and composite risk estimates of small absolute benefits for those at lowest distant recurrence risk [20]. For the revised BCI low-risk group in SOFT, the 10-year distant recurrence estimates was 7.2 % (95 %CI 4.0–13.1 %) for the SOFT tamoxifen control group and 2.7 % (95 % CI 1.4–5.3 %) for the OFS-containing treatment groups, implying a magnitude of risk and potential benefit sufficient to consider OFS-containing endocrine therapy regimens [20].

The 70-gene assay (MammaPrint) ultra-low risk group has been discussed relative to the BCI minimal-risk group in the context of postmenopausal women [15,16]. In the MINDACT trial, 15 % of women ≤50 years at diagnosis were ultra-low risk (n = 329) and the 8-year DRFI was 95.6 % (95 % CI 93.3–98.0) [21].

The additional BCI cutpoint was identified and validated to classify postmenopausal patients with HR+, HER2-negative or positive, node-negative breast cancer with minimal (<5 %) risk of distant recurrence within 10 years [15,16]. This study extends the clinical validity of BCI minimal-risk classification in the settings of adjuvant tamoxifen and OFS-containing adjuvant endocrine therapy regimens, aimed at facilitating discussions of OFS efficacy and side effects trade-offs.

The study has limitations. The prognostic validity is assessed in the setting of the adjuvant therapies received by SOFT and TEXT participants. More than one-third of patients received adjuvant chemotherapy, including 15 % and 27 % of the minimal-risk subgroups, respectively, and OFS was given to all TEXT patients and two-thirds SOFT patients. The minimal-risk group comprised approximately 20 % of all patients, which although similar to postmenopausal patient cohorts previously studied, was less than 400 patients studied, of whom only 62 were assigned to tamoxifen without OFS. The confidence intervals for 10-year incidence of distant recurrence in the minimal-risk subgroups remain wide. Further evaluation of the BCI minimal- and low-risk differentiation in a large set of premenopausal patients with clinical-pathological low-risk (e.g., stage I, grade 1/2) tumors and more diverse chemo-endocrine therapy would be of interest.

In conclusion, this study validated the prognostic ability of the adjusted BCI model minimal-risk cutpoint to re-classify a subset of premenopausal patients with HR+ N0 early breast cancer estimated to have minimal risk of distant recurrence within 10 years. These findings suggest that patients with tumors categorized as minimal-risk may derive only a small benefit from OFS-containing endocrine therapy regimens and may represent a subgroup warranting a personalized decision about the potential benefit versus side effects of OFS and adjuvant tamoxifen alone as endocrine therapy.

## CRediT authorship contribution statement

**Ruth M. O'Regan:** Writing – review & editing, Writing – original draft, Conceptualization. **Yue Ren:** Writing – review & editing, Writing – original draft, Formal analysis. **Yi Zhang:** Writing – review & editing, Writing – original draft, Methodology, Conceptualization. **Gini F. Fleming:** Writing – review & editing, Resources, Investigation. **Prudence A. Francis:** Writing – review & editing, Investigation. **Olivia Pagani:** Writing – review & editing, Resources, Investigation. **Barbara A. Walley:** Writing – review & editing, Resources, Investigation. **Roswitha Kammler:** Writing – review & editing, Project administration. **Patrizia Dell’Orto:** Writing – review & editing, Investigation. **Giuseppe Viale:** Writing – review & editing, Supervision, Investigation. **Sherene Loi:** Writing – review & editing, Supervision, Resources. **Marco Colleoni:** Writing – review & editing, Supervision, Resources. **Kai Treuner:** Writing – review & editing, Writing – original draft, Methodology, Conceptualization. **Meredith M. Regan:** Writing – review & editing, Writing – original draft, Validation, Investigation, Conceptualization.

## Data statement

After publication, access to deidentified participant data may be requested by researchers by submitting a proposal (to stat_center@ ibcsg.org), which will be reviewed for scientific merit and feasibility in accordance with the Guidelines for Collaborative research (https://www.ibcsg.org/images/Member/Publi/Documents/Guidelines_for_Collaborative_Research_for_ETOP_IBCSG_Partners_Foundation_Dec_2022.pdf) and data sharing policy (https://www.ibcsg.org/images/Member/Publi/Documents/Data_Sharing_Policy_for_IBCSG_Trials_Dec_2022.pdf) for IBCSG trials.

## Funding

This translational research study was funded by Biotheranostics, Inc., a Hologic company.

The TEXT and SOFT clinical trials have received financial support for trial conduct including long-term follow-up from Pfizer, the International Breast Cancer Study Group, the Breast Cancer Research Foundation (16-185, 17-187, 18-003, 19-011, 20-011, 21-011, 22-011, 23-011, to MMR), Ipsen, DebioPharm, TerSera, AstraZeneca and private donors. Pfizer and Ipsen provided drug supply. Support for central pathology included Susan G. Komen for the Cure Promise Grant [grant number KG080081 to OP, GV, MMR] and the Breast Cancer Research Foundation. Additional support for the IBCSG: Frontier Science Foundation, Swiss Group for Clinical Cancer Research (SAKK), Oncosuisse, Cancer League Switzerland, Foundation for Clinical Cancer Research of Eastern Switzerland (OSKK), US National Cancer Institute [US NIH grant number CA075362].

Grant support of cooperative groups for TEXT and SOFT conduct: In Australia & New Zealand, support from Breast Cancer Trials Australia & New Zealand [NHMRC grant numbers 351161, 510788, 1105058] and Breast Cancer Research Foundation for biobank. In the US and Canada, support by the US National Cancer Institute of the National Institutes of Health via the Alliance for Clinical Trials in Oncology [US NIH grant number U10CA180821]; SWOG [US NIH grant number U10CA180888, UG1CA233160, UG1CA233329]; ECOG-ACRIN Cancer Research Group [US NIH grant numbers U10CACA180820,U10CA180794]; NRG Oncology [US NIH grant numbers U10CA180868, U10CA180822, UG1CA189867]; Canadian Cancer Trials Group [US NIH grant number U10CA180863]; and Canadian Cancer Society [grant number707213)]. The content is solely the responsibility of the authors and does not necessarily represent the official views of the US National Institutes of Health.

## Declaration of competing interest

**RO** serves as an advisor for Biotheranostics. **YZ** and **KT** are employed at Biotheranostics, a Hologic Company. **YZ** and **KT** are owners of Biotheranostics, a Hologic Company, stock. **YZ** reports an issued patent for the Breast Cancer Index (BCI) test as well as another one pending. **GFF** reports serving as the institutional PI for trials with the following sponsors: Roche, Iovance, Sermonix, Compugen, AstraZeneca, Astellas, K Group Beta, Pfizer, Artios, Blueprint, Duality Bio and PHEAST. **PAF** reports receiving an honorarium from Eli Lilly (for a lecture). **SL** reports receiving research funding (payments to institution) from Novartis, Bristol Myers Squibb, MSD, Pfizer, Eli Lilly, Nektar Therapeutics, Gilead Sciences, Astra Zeneca/Daiichi Sankyo and Roche-Genentech; reports serving as a consultant (paid and unpaid) to Novartis, Bristol Myers Squibb, MSD, AstraZeneca/Daiichi Sankyo, Eli Lilly, Amaroq Therapeutics, Mersana Therapeutics, Domain Therapeutics, BioNTech, Bicycle Therapeutics, Exact Sciences, Menari Asia-Pacific, SAGA Diagnostics, Gilead Sciences, Roche-Genentech, and Adanate; receiving payment of honoraria for speaking at educational events from Novartis, MSD, Gilead Sciences, Exact Sciences, Roche-Genentech, and AstraZeneca; receiving support for attending meetings and/or travel (payments to institution) from BMS and Roche-Genentech; as well as reports serving as the Co-Chair for the International Breast Cancer Study Group (IBCSG) Scientific Committee. **MC** reports serving as the Co-Chair for the International Breast Cancer Study Group (IBCSG) Scientific Committee. **KT** reports a pending patent for the BCI test. **MMR** reports receiving grant support (to institution) from: Biotheranostics, DebioPharm, Ipsen, TerSera Therapeutics, AstraZeneca, and Pfizer; receiving personal fees from Tolmar and TerSera Therapeutics; having an advisory role with Ipsen and DebioPharm; receiving grant support (payments to institution outside of submitted work) from BMS, Novartis, Pfizer, and Roche, and receiving personal fees from BMS outside of submitted work. All remaining authors have declared no conflicts of interest (**YR, OP, BAW, RK**, **PD**, **GV)**

## Abbreviations:

BCI: Breast Cancer Index
cDNA: Complementary DNA
CI: Confidence interval
DR: Distant recurrence
DRFI: Distant recurrence-free interval
FFPE: Formalin-fixed paraffin-embedded
HER2: Human epidermal growth factor receptor 2
HR: Hazard ratio
HR+: Hormone-receptor-positive
N0: Lymph node-negative
OFS: Ovarian function suppression
PCR: Reverse Transcription polymerase chain reaction
REMARK: Reporting recommendations for tumour Marker prognostic studies
RT-PCR: Polymerase chain reaction
SOFT: Suppression of Ovarian Function Trial
TEXT: Tamoxifen and Exemestane Trial
