# Supplementary material for: Identifying premenopausal patients with early-stage hormone receptor–positive breast cancer at minimal risk of distant recurrence by breast cancer index
Source: Breast. 2026 Jan 29;86:104714. doi: 10.1016/j.breast.2026.104714 (PMC12887762; doi:10.1016/j.breast.2026.104714)
Supplement: Multimedia component 1 [file mmc1.docx]

**SUPPLEMENTARY TABLES**

**Table S1**. Clinicopathologic characteristics of all patients with lymph node-negative (N0) breast cancers enrolled in SOFT (n=1995 of 3047), of the 1110-patient N0 BCI analysis cohort subset according to adjusted BCI model risk classifications, and of those 885 patients with N0 cancers for whom BCI was not determined.

|  |  | | **SOFT BCI Analysis Cohort with N0 Breast Cancers** | | | | | | | |  | |
| --- | --- | --- | --- | --- | --- | --- | --- | --- | --- | --- | --- | --- |
|  | SOFT  All N0 | | BCI high risk | | BCI intermediate risk | | BCI (revised) low risk | | BCI minimal risk | | SOFT N0  no BCI | |
|  | n | % | n | % | n | % | n | % | n | % | n | % |
| *Patients in* ***SOFT*** *analysis population* | *1995* | *100.0* | *193* | *100.0* | *240* | *100.0* | *480* | *100.0* | *197* | *100.0* | *885* | *100.0* |
| Treatment assignment |  |  |  |  |  |  |  |  |  |  |  |  |
| T | 662 | 33.2 | 67 | 34.7 | 99 | 41.3 | 155 | 32.3 | 62 | 31.5 | 279 | 31.5 |
| T+OFS | 662 | 33.2 | 63 | 32.6 | 67 | 27.9 | 155 | 32.3 | 68 | 34.5 | 309 | 34.9 |
| E+OFS | 671 | 33.6 | 63 | 32.6 | 74 | 30.8 | 170 | 35.4 | 67 | 34.0 | 297 | 33.6 |
| Chemotherapy |  |  |  |  |  |  |  |  |  |  |  |  |
| No | 1294 | 64.9 | 64 | 33.2 | 140 | 58.3 | 343 | 71.5 | 168 | 85.3 | 579 | 65.4 |
| Yes | 701 | 35.1 | 129 | 66.8 | 100 | 41.7 | 137 | 28.5 | 29 | 14.7 | 306 | 34.6 |
| Age at randomization |  |  |  |  |  |  |  |  |  |  |  |  |
| <35 | 158 | 7.9 | 31 | 16.1 | 27 | 11.3 | 29 | 6.0 | 5 | 2.5 | 66 | 7.5 |
| 35-39 | 294 | 14.7 | 50 | 25.9 | 31 | 12.9 | 76 | 15.8 | 16 | 8.1 | 121 | 13.7 |
| 40-44 | 592 | 29.7 | 59 | 30.6 | 81 | 33.8 | 121 | 25.2 | 54 | 27.4 | 277 | 31.3 |
| 45-49 | 704 | 35.3 | 43 | 22.3 | 80 | 33.3 | 176 | 36.7 | 83 | 42.1 | 322 | 36.4 |
| 50+ | 247 | 12.4 | 10 | 5.2 | 21 | 8.8 | 78 | 16.3 | 39 | 19.8 | 99 | 11.2 |
| Tumor size |  |  |  |  |  |  |  |  |  |  |  |  |
| <=2 cm | 1530 | 76.7 | 114 | 59.1 | 174 | 72.5 | 367 | 76.5 | 169 | 85.8 | 706 | 79.8 |
| >2 cm | 437 | 21.9 | 78 | 40.4 | 64 | 26.7 | 108 | 22.5 | 28 | 14.2 | 159 | 18.0 |
| Unknown | 28 | 1.4 | 1 | 0.5 | 2 | 0.8 | 5 | 1.0 | . | . | 20 | 2.3 |
| Tumor grade |  |  |  |  |  |  |  |  |  |  |  |  |
| 1 | 608 | 30.5 | 12 | 6.2 | 42 | 17.5 | 173 | 36.0 | 100 | 50.8 | 281 | 31.8 |
| 2 | 1040 | 52.1 | 82 | 42.5 | 141 | 58.8 | 264 | 55.0 | 87 | 44.2 | 466 | 52.7 |
| 3 | 313 | 15.7 | 96 | 49.7 | 57 | 23.8 | 34 | 7.1 | 8 | 4.1 | 118 | 13.3 |
| Unknown | 34 | 1.7 | 3 | 1.6 | . | . | 9 | 1.9 | 2 | 1.0 | 20 | 2.3 |
| HER2 status* |  |  |  |  |  |  |  |  |  |  |  |  |
| Negative | 1738 | 87.1 | 146 | 75.6 | 206 | 85.8 | 448 | 93.3 | 184 | 93.4 | 754 | 85.2 |
| Positive | 196 | 9.8 | 39 | 20.2 | 28 | 11.7 | 24 | 5.0 | 9 | 4.6 | 96 | 10.8 |
| Unknown/Not done | 61 | 3.1 | 8 | 4.1 | 6 | 2.5 | 8 | 1.7 | 4 | 2.0 | 35 | 4.0 |

*Note, patients with HER2-positive disease did not all receive (neo)adjuvant HER2-targeted therapy based upon the enrollment period beginning prior to regulatory approval of trastuzumab.

**Table S2**. Clinicopathologic characteristics of all patients with lymph node-negative (N0) breast cancers enrolled in TEXT (n=1377 of 2660), of the 1377-patient N0 BCI analysis cohort subset according to adjusted BCI model risk classifications, and of those 462 patients with N0 cancers for whom BCI was not determined.

|  |  | | **TEXT BCI Analysis Cohort with N0 Breast Cancers** | | | | | | | |  | |
| --- | --- | --- | --- | --- | --- | --- | --- | --- | --- | --- | --- | --- |
|  | TEXT  All N0 | | BCI high risk | | BCI intermediate risk | | BCI (revised) low risk | | BCI minimal risk | | TEXT N0  no BCI | |
|  | n | % | n | % | n | % | n | % | n | % | n | % |
| *Patients in* ***TEXT*** *analysis population* | *1377* | *100.0* | *162* | *100.0* | *224* | *100.0* | *350* | *100.0* | *179* | *100.0* | *462* | *100.0* |
| Treatment assignment |  |  |  |  |  |  |  |  |  |  |  |  |
| T+OFS | 686 | 49.8 | 81 | 50.0 | 116 | 51.8 | 172 | 49.1 | 90 | 50.3 | 227 | 49.1 |
| E+OFS | 691 | 50.2 | 81 | 50.0 | 108 | 48.2 | 178 | 50.9 | 89 | 49.7 | 235 | 50.9 |
| Chemotherapy |  |  |  |  |  |  |  |  |  |  |  |  |
| No | 835 | 60.6 | 57 | 35.2 | 119 | 53.1 | 239 | 68.3 | 130 | 72.6 | 290 | 62.8 |
| Yes | 542 | 39.4 | 105 | 64.8 | 105 | 46.9 | 111 | 31.7 | 49 | 27.4 | 172 | 37.2 |
| Age at randomization |  |  |  |  |  |  |  |  |  |  |  |  |
| <35 | 101 | 7.3 | 16 | 9.9 | 22 | 9.8 | 22 | 6.3 | 5 | 2.8 | 36 | 7.8 |
| 35-39 | 198 | 14.4 | 33 | 20.4 | 40 | 17.9 | 35 | 10.0 | 25 | 14.0 | 65 | 14.1 |
| 40-44 | 457 | 33.2 | 53 | 32.7 | 72 | 32.1 | 144 | 41.1 | 58 | 32.4 | 130 | 28.1 |
| 45-49 | 489 | 35.5 | 47 | 29.0 | 72 | 32.1 | 124 | 35.4 | 70 | 39.1 | 176 | 38.1 |
| 50+ | 132 | 9.6 | 13 | 8.0 | 18 | 8.0 | 25 | 7.1 | 21 | 11.7 | 55 | 11.9 |
| Tumor size |  |  |  |  |  |  |  |  |  |  |  |  |
| <=2 cm | 998 | 72.5 | 87 | 53.7 | 157 | 70.1 | 258 | 73.7 | 144 | 80.4 | 352 | 76.2 |
| >2 cm | 369 | 26.8 | 75 | 46.3 | 66 | 29.5 | 89 | 25.4 | 35 | 19.6 | 104 | 22.5 |
| Unknown | 10 | 0.7 | . | . | 1 | 0.4 | 3 | 0.9 | . | . | 6 | 1.3 |
| Tumor grade |  |  |  |  |  |  |  |  |  |  |  |  |
| 1 | 285 | 20.7 | 5 | 3.1 | 17 | 7.6 | 81 | 23.1 | 62 | 34.6 | 120 | 26.0 |
| 2 | 794 | 57.7 | 69 | 42.6 | 145 | 64.7 | 222 | 63.4 | 106 | 59.2 | 252 | 54.5 |
| 3 | 288 | 20.9 | 88 | 54.3 | 62 | 27.7 | 45 | 12.9 | 9 | 5.0 | 84 | 18.2 |
| Unknown | 10 | 0.7 | . | . | . | . | 2 | 0.6 | 2 | 1.1 | 6 | 1.3 |
| HER2 status |  |  |  |  |  |  |  |  |  |  |  |  |
| Negative | 1197 | 86.9 | 117 | 72.2 | 187 | 83.5 | 325 | 92.9 | 166 | 92.7 | 402 | 87.0 |
| Positive | 169 | 12.3 | 45 | 27.8 | 37 | 16.5 | 22 | 6.3 | 11 | 6.1 | 54 | 11.7 |
| Unknown/Not done | 11 | 0.8 | . | . | . | . | 3 | 0.9 | 2 | 1.1 | 6 | 1.3 |
